# A study on the association of serum 1,5-anhydroglucitol levels and the hyperglycaemic excursions as measured by continuous glucose monitoring system among people with type 2 diabetes in China

**DOI:** 10.1002/dmrr.2278

**Published:** 2012-05-11

**Authors:** Y Wang, Y L Zhang, Y P Wang, C H Lei, Z L Sun

**Affiliations:** 1 Department of Endocrinology, Zhongda Hospital, Southeast UniversityNanjing, China; 2 Institute of Diabetes, Medical School, Southeast UniversityNanjing, China

**Keywords:** 1,5-anhydroglucitol, type 2 diabetes mellitus, hyperglycaemic excursions

## Abstract

**Background:**

Blood glucose excursion is an important component of the glycaemic burden, but there are no indexes that can directly reflect them. The aim was to evaluate the values and significance of serum 1,5-anhydroglucitol (1,5-AG) in people with type 2 diabetes mellitus in China and to elucidate the relationship between 1,5-AG and traditional indexes of glycaemic excursions by continuous glucose monitoring.

**Methods:**

A total of 576 healthy adults and 292 patients were included, and their 1,5-AG, fasting blood glucose and postprandial blood glucose and glycated haemoglobin were measured. For the 34 patients, their mean blood glucose, standard deviation of blood glucose, mean amplitude of glucose excursion, mean of daily differences, low blood glucose M-value index and the area under the curve for blood glucose above 180 mg/dL were calculated by use of a continuous glucose monitoring system.

**Results:**

Serum levels of 1,5-AG among healthy adults were 28.44 ± 8.76 µg/mL with a significant gender bias rather than age bias. The 1,5-AG levels in people with type 2 diabetes mellitus were 4.57 ± 3.71 µg/mL, which were lower than those seen in the healthy adults. There was a correlation between 1,5-AG and glycated haemoglobin, fasting blood glucose, and postprandial blood glucose (*r* = −0.251, −0.195 and −0.349, respectively; all had *p* < 0.05). The continuous glucose monitoring system demonstrated that 1,5-AG presents a negative correlation with mean blood glucose, standard deviation of blood glucose, mean amplitude of glucose excursion and mean of daily differences for 7 days and with the area under the curve for blood glucose above 180 mg/dL on the third, fourth and seventh days.

**Conclusions:**

1,5-AG may serve as a marker of hyperglycaemia and 7-day hyperglycaemic excursions as well as being a useful adjunct to glycated haemoglobin for blood glucose monitoring in patients with diabetes. Copyright © 2012 John Wiley & Sons, Ltd.

## Introduction

Because of the rapid lifestyle changes in China, there is a concern that diabetes may become an epidemic. According to the latest information available, the prevalence of total diabetes in China is as high as 9.7%, accounting for 92.4 million adults with diabetes. These results indicate that diabetes has become a major public health problem in China [1].

It is well known that poor control of blood glucose may lead to both microvascular and macrovascular complications that are correlated with hyperglycaemia and blood glucose excursions [2,3]. Glucose excursions, especially in the postprandial state, are independent risk factors for macrovascular complications of diabetes mellitus [4–6]. In current clinical practices, the common indexes to evaluate the blood glucose states are glycated haemoglobin (HbA_1c_) and fructosamine (FA). However, these can only reflect the integrated average blood glucose concentration of the preceding 8–12 weeks or 2–3 weeks, respectively, and potentially overlook the important hyperglycaemic excursions that may be balanced out by hypoglycaemia. Thus, neither HbA_1c_ nor FA can reflect recent glycaemic excursions sensitively. So, in addition to HbA_1c_ and FA, there is an imperative need for more intensive and sensitive blood glucose monitoring markers to reveal not only blood glucose levels but also recent hyperglycaemic excursions. This information will facilitate adjusting diabetic treatment effectively and help to create more reasonable therapeutic regimens.

1,5-anhydroglucitol (1,5-AG) was discovered in humans in 1972 [7]. It is reported that 99.9% of the filtered 1,5-AG in the kidney is reabsorbed in the renal tubules, and the concentrations of 1,5-AG in the bloodstream are fairly constant because of the balance between intake and urinary excretion. When blood glucose is higher than the renal threshold for glucose, renal reabsorption of 1,5-AG is competitively inhibited by glucose in the renal tubule, and subsequently, the serum level of 1,5-AG decreases [8,9]. When blood glucose levels return to baseline, the reabsorption of 1,5-AG is restored, and the blood levels of 1,5-AG return to baseline values. Some studies have verified that 1,5-AG values are sensitive to the changes in blood glucose and can reflect even transient elevations of glycaemia within a few days.

With advancements in blood glucose measurement techniques, the continuous glucose monitoring system (CGMS) seems to be the most useful device in obtaining a comprehensive look at glycaemic status [10]. However, the CGMS, in fact, requires extensive training to use and can be somewhat complicated for the average person with diabetes to use. Additionally, the CGMS are also very expensive and would likely not be used widely.

To evaluate the significance of 1,5-AG in Chinese people with diabetes, we undertook this large scale study to investigate and confirm the reference range of 1,5-AG levels in both healthy Chinese adults and patients with type 2 diabetes mellitus (T2DM) and to examine the relationship of 1,5-AG levels with the parameters of hyperglycaemic excursion using CGMS.

## Patients and methods

### Research subjects

A total of 576 healthy adults underwent health examination at ZhongDa Hospital, affiliated to the Southeast University, Nanjing, China, between January and December 2010 and were assigned to a control group. Two hundred fifty-four were men and 322 were women. The subjects ranged in age from 22 to 78 (45.6 ± 16.5) years old. A detailed medical examination was taken to demonstrate that none of the control subjects had diabetes mellitus, and their blood glucose, urinary glucose, liver function and renal function were all within normal range [A fasting blood glucose (FBG) level of less than 6.1 mmol/L and postprandial blood glucose (PBG) level less than 7.8 mmol/L were considered to be normal according to the recommendations of the World Health Organization and the International Diabetes Federation.

Two hundred and ninety-two patients with T2DM, who had come for consultation to the hospital between January and December 2010, comprised the T2DM group. In this group, 34 patients were monitored continuously for glucose parameters for 7 days by using CGMS. Of the subjects in this group, 140 were men and 152 were women. These subjects ranged in age from 28 to 69 (48.4 ± 6.7) years old. Most of them are overweight [body mass index 24.8 ± 4.7 kg/m^2^], and the concentrations of glutamic acid decarboxylase, insulin and islet cell antibodies in their blood were all negative. The blood concentration of insulin was 15.2 ± 4.7 mU/L for fasting blood glucose. They had normal kidney function (blood creatinine levels were less than 132 µmol/L or urinary albumin excretion rates were less than 20 µg/min) and normal liver function. Exclusion criteria for this study included diabetic ketoacidosis, hyperosmolar coma, hypoglycaemic coma, diabetic nephropathy, renal insufficiency, liver dysfunction, patients in gestational period and patients requiring long-term total parenteral nutrition within the previous month.

Approval to conduct the trial was obtained from the local Research and Ethics Committees, and all participants provided written informed consent.

### Measurement of blood glucose indexes

Every subject was required to fast for at least 8 h. The following morning, 2 mL of venous blood, from the ulnar vein of each subject, was drawn into vacutainers and shaken gently to mix the additives with the blood. The blood samples were also taken from these subjects 2 h after they consumed 75 g of glucose. The samples were subjected to centrifugation, at 3000 rpm for 5 min at room temperature, and the supernatant serum was utilized to test FBG and PBG. Additionally, samples were stored at −80 °C prior to measurement of 1,5-AG. HbA_1c_ was determined from whole blood samples via a turbidimetric inhibition immunoassay (Tina-Quant; Roche Diagnostics), automated on the Hitachi 917. Serum 1,5-AG was measured with the GlycoMark assay (Tomen America, New York, NY), which was automated on a Hitachi 917 analyzer (Roche Diagnostics, Indianapolis, IN).

CGMS [11] was designed and developed by Medtronic (Medtronic MiniMed) and has been approved for clinical application in the USA. Capillary measurements were obtained four times daily for validation of CGMS values.

### Evaluating indicators

MBG: mean values of blood glucose monitored by CGMS, 288 times per day for 7 days.

SDBG: standard deviation of mean levels of blood glucose monitored by CGMS, 288 times per day for 7 days.

MAGE: mean amplitude of glucose excursion. Statistics were applied to express the glycaemic excursions that were more than one standard deviation away from the mean, during the time of observation in subjects wearing CGMS monitoring equipment. The amplitude of glycaemic excursion from the direction of the peak to the valley was utilized to calculate the drift rate. MAGE values were average values for all the mean amplitudes of glycaemic excursion [12].

MODD: mean of daily differences. Inter-day glycaemic variation was assessed using the MODD. The absolute value of the difference between glucose values, taken on two consecutive days at the same time, was calculated. The MODD is the mean of these differences [13]. All readings of the trace, where there is a reading 24 h previously, are included in the calculation of the MODD.

LBMI: low blood glucose M-value index. This is a composite score reflecting the frequency and extent of low blood glucose. It accounts for 40%–60% of the variance of future severe hypoglycaemic episodes within the following 3–6 months [14].

AUC180: This indicates the area under the curve for values that are greater than or equal to 180 mg/dL of blood glucose. CGMS software can directly demonstrate the values on the third, fourth and seventh days.

### Statistical analysis

Statistical analyses were performed using the social sciences (SPSS13.0). All values were expressed as 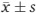. The statistical significance of differences was analysed by the Student's *t*-test, Chi-square test or one-way analysis of variance. Pearson's coefficient was used for the correlation analysis. Statistical significance was determined at *p*-values <0.05.

## Results

### Range of 1,5-AG levels in healthy adults

**Figure 1:**
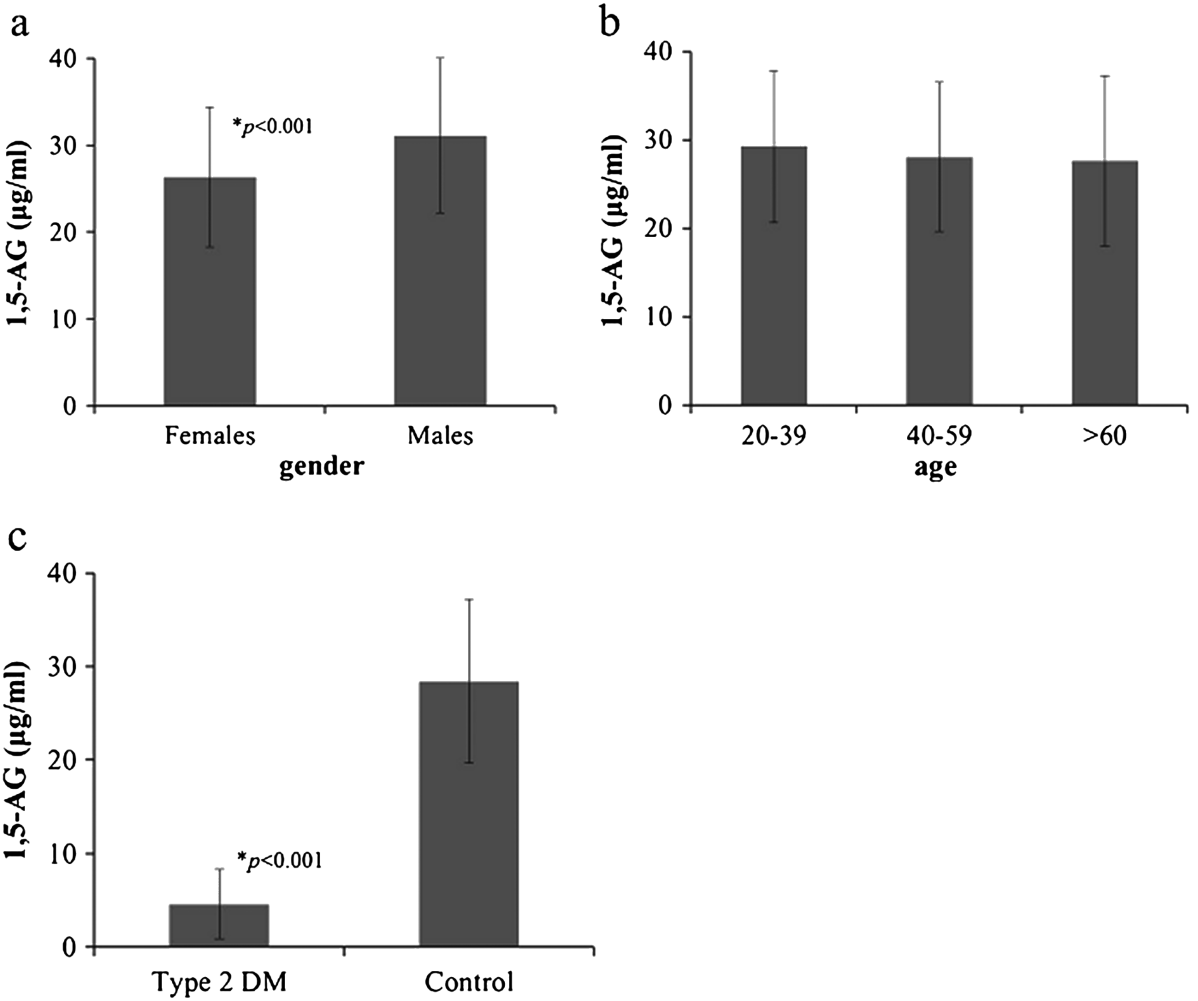
The characteristics of 1,5-anhydroglucitol (1,5-AG) levels in healthy adults. 1,5-AG concentrations in the control group were 28.44 ± 8.76 µg/mL. (a) when compared with different genders, concentrations of men (31.11 ± 8.91 µg/mL ) were higher than that of women (26.33 ± 8.05 µg/mL) ( *p* < 0.001); (b) when compared with different age groups, there was no significant difference ( 20–39 years old: 29.21 ± 8.54 µg/mL, 40–59 years old: 28.06 ± 8.46 µg/mL, >60 years old: 27.57 ± 9.62 µg/mL ) ( *p* = 0.176) and (c) 1,5-AG concentrations in the control group were significantly higher than in type 2 diabetes mellitus group 4.57 ± 3.71 µg/mL, (*p* < 0.001)

Overall, the 1,5-AG values of 576 healthy adults were found to be consistent with a normal distribution. According to 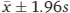, the normal reference range is calculated as 11.27–45.61 µg/mL (28.44 ± 8.76 µg/mL). The results of the analyses show that there is a significant gender bias but not a statistically significant age bias (Figure 1).

### Range of 1,5-AG level in patients with T2DM

The clinical characteristics of 292 patients with T2DM and 34 of them with CGMS are summarized in Table 1. In this study, the serum 1,5-AG concentration of patients with T2DM (4.57 ± 3.71 µg/mL) was significantly lower than that observed in the control group (Figure 1).

**Table 1:** Clinical characteristics of 292 type 2 diabetic patients and 34 type 2 diabetic patients with continuous glucose monitoring system

	N	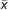 ± *s*
FBG(mmol/L)	292	8.79 ± 3.47
PBG(mmol/L)	292	14.14 ± 3.36
HbA_lc_(%)	292	8.64 ± 1.65
MBG(mmol/L)	34	7.70 ± 1.25
SDBG(mmol/L)	34	1.83 ± 0.76
MAGE(mmol/L)	34	4.56 ± .50
MODD(mmol/L)	34	1.79 ± 0.83
LBMI	34	25.35 ± 22.90

FBG; fasting blood glucose, PBG; postprandial blood glucose, HbA_1c_; glycated haemoglobin, MBG; meanblood glucose, SDBG; standard deviation of blood glucose, MAGE; mean amplitude of glucose excursion, MODD; mean of daily differences, LBMI; low blood glucose M-value index.

### Correlation between 1,5-AG and blood glucose characteristics of patients with T2DM

**Figure 2:**
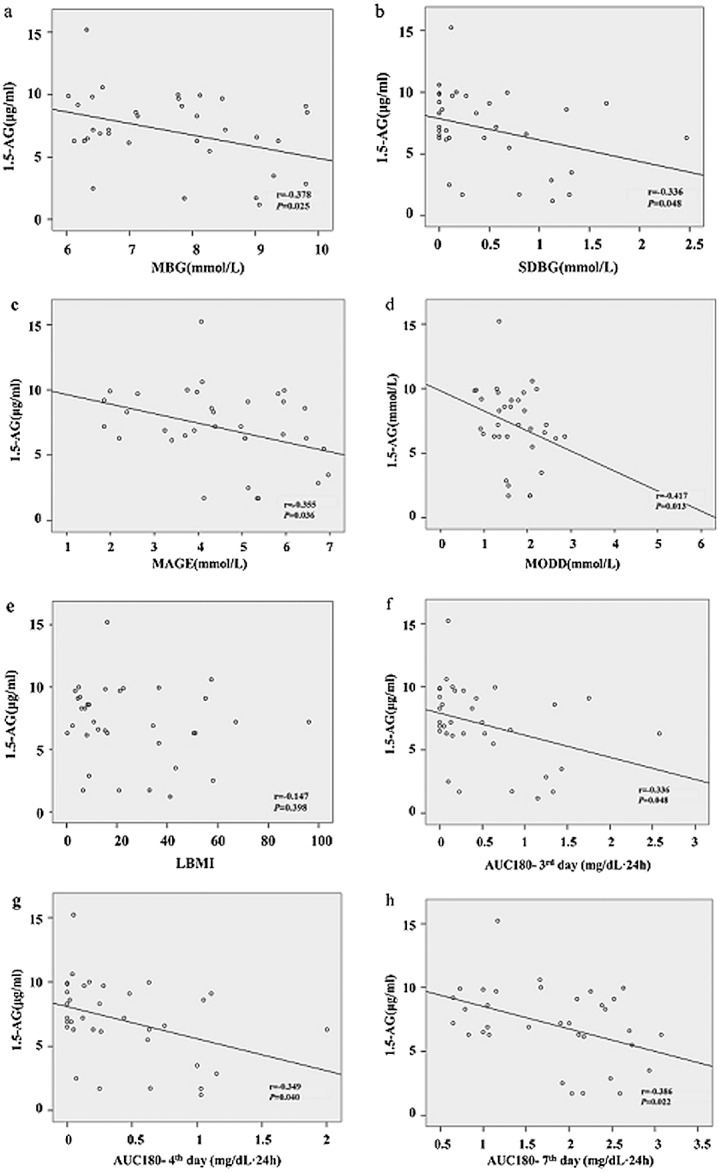
The correlations between 1,5-anhydroglucitol (1,5-AG) and blood glucose characteristics of patients with type 2 diabetes mellitus. 1,5-AG levels were negatively correlated with (a) mean blood glucose (MBG); (b) standard deviation of blood glucose (SDBG); (c) mean amplitude of glucose excursion (MAGE); (d) mean of daily differences (MODD); (f) area under the curve for blood glucose above 180 mg/dL-3rd day (AUC180 -3rd); (g) area under the curve for blood glucose above 180 mg/dL-4th day (AUC180-4th); (h) area under the curve for blood glucose above 180 mg/dL-7th day (AUC180-7th) and was not correlated with (e) low blood glucose M-value index (LBMI). Glycated haemoglobin levels were not correlated with MBG, SDBG, MAGE, MODD, LBMI, and AUC180-3rd, AUC180-4th, AUC180-7th (the figures were not shown)

We evaluated the correlation between 1,5-AG and characteristics of patients with T2DM including FBG, PBG and HbA_1c_ and found that in 292 patients, 1,5-AG levels were negatively correlated with FBG, PBG and HbA_1c_ (*r* = −0.251, −0.195 and −0.349, respectively; *p* < 0.001). Furthermore, in the 34 patients monitored with CGMS, the 1,5-AG levels were negatively correlated with levels of MBG, SDBG, MAGE, MOOD, AUC180-3rd, AUC180-4th and AUC180-7th (*r* = 0.378, 0.336, 0.355, 0.417, 0.336, 0.349, 0.386, respectively; *p* < 0.05). HbA_1c_ levels were not correlated with any of the aforementioned measured parameters (*r* = 0.278, 0.152, 0.250, 0.100, 0.119, 0.175, 0.224, respectively; *p* > 0.100) (Figure 2).

LBMI levels were not found to have any correlation with 1,5-AG levels or HbA_1c_ levels (*r* = 0.147 and 0.207, respectively; *p* > 0.100 ).

## Discussion

The present study determined the test values of serum 1,5-AG in healthy adults and in patients with T2DM in China. We demonstrate that in healthy Chinese adults, the level of 1,5-AG is 28.44 ± 8.76 µg/mL (ranges from 11.27 to 45.61 µg/mL), which is consistent with three previous clinical studies conducted in Japan, showing normal ranges of 1,5-AG at 18.7–35.3, 23.8 ± 7.2 and 9.6–38.8 µg/mL [8,9,15]. Our results were also comparable with a US study, where the average range of 1,5-AG was 15.6–29.2 µg/mL [16]. In a small study in Shanghai, China in 1999, the 57 subjects had an average FPG level of 4.81 ± 0.52 mmol/L and the 1,5-AG level was 27.96 ± 7.75 µg/mL [17]; the result was in an accordance with our study.

Our 1,5-AG data also showed that there was a statistically significant difference between genders, with the 1,5-AG level higher among men than women. These results are consistent with a foreign study and may be attributable to the physical differences in renal excretion rate between genders [16]. However, a different result seen in another study may result from their small sample sizes [18]. In general opinion, the absolute range of the reference interval in all healthy populations is quite similar [16].

In our study, the average 1,5-AG concentration from 292 Chinese patients with T2DM was 4.57 ± 3.71 µg/mL, significantly lower than that seen in healthy adults (*p* < 0.001). Studies from Poland, the USA and Japan also reported the same lower 1,5-AG concentrations in patients with diabetes mellitus when compared with healthy subjects [19–21]. The lower 1,5-AG concentration observed in Chinese patients with T2DM has a negative correlation with hyperglycaemia (with FBG, PBG and HbA_1c_ *r* = −0.251, −0.195, −0.349, respectively; *p* < 0.001).

In conclusion, according to these results, we have demonstrated that the 1,5-AG values in diabetic patients were distinctly different from that seen in healthy individuals in China, and these values had a negative correlation with hyperglycaemic index. We deduced that the lower value of 1,5-AG may also be a marker of the status of hyperglycaemia in Chinese patients with T2DM.

Kishimoto M reported that 1,5-AG could be a useful index of glycaemic excursions in patients with reasonably well-controlled diabetes (mean HbA_1c_: 7.1%) [22]. The 1,5-AG marker reflects glycaemic excursions, often in the postprandial hyperglycaemic state, more accurately than HbA_1c_ or FA [20]. This demonstrated that 1,5-AG was not only a marker for the state of hyperglycaemia but also for postprandial hyperglycaemia in studies reported from certain foreign countries.

In our test group, 34 patients with T2DM were monitored with CGMS consecutively for 7 days, and the data showed many useful parameters: SDBG, MAGE, MODD and LBMI. These data indicated that the 7-day average level of 1,5-AG had a negative correlation not only with MBG, which indicates a high blood glucose status, but also with SDBG, MAGE and MODD. Additionally, glycaemic test values of AUC180, obtained from CGMS, indicated that the 1,5-AG level had negative correlations with AUC180-3rd, AUC180-4th and AUC180-7th, which implies fluctuation of high blood glucose. 1,5-AG did not show a significant association with LBMI, which was a composite score reflecting the frequency and extent of low blood glucose. This may indicate that 1,5-AG levels are not associated with the events of hypoglycaemia.

In Chinese patients with T2DM, low 1,5-AG levels were associated with poorly controlled glucose (average HbA_1c_ is 8.6 ± 1.6%). 1,5-AG levels may also reflect the extent of daily hyperglycaemic excursions for the previous 3–7 days. In contrast, HbA_1c_ had no correlation with MBG, SDBG, MAGE, MODD, AUC180-3rd, AUC180-4th or AUC180-7th (*r* = 0.278, 0.152, 0.250, 0.100, 0.119, 0.175, 0.224, respectively; *p* > 0.100). In the present study, we also showed that HbA_1c_ could not reflect the recent hyperglycaemic excursions in Chinese patients with T2DM.

According to the metabolic characteristics of 1,5-AG, we know that the normal concentration of 1,5-AG is stable, and in lab testing, there is no special requirement with regard to the test specimen, for example both serum or plasma can be tested for 1,5-AG level. Specimens can be stored for a long duration (at 4 °C for 4 weeks and −20 °C for 2 years) and can be evaluated for clinical implications [23]. This makes it easy and convenient for a clinical practice to undertake clinical testing situations.

In conclusion, 1,5-AG can be a marker of hyperglycaemia and blood glucose excursions for 3–7 days in Chinese patients with T2DM. If 1,5 AG measurements can be used regularly, in addition to HbA_1c_ and FA, it would be extremely helpful for adjusting treatment remedies to reduce the hazards that occur with hyperglycaemia and the hyperglycaemic excursions and ultimately effectively improve the prognosis of patients with T2DM.

## References

[1] Yang W, Lu J, Weng J (2010). Prevalence of diabetes among men and women in China. N Engl J Med.

[2] Hanefeld M, Fischer S, Julius U (1996). Risk factors for myocardial infarction and death in newly detected NIDDM: the Diabetes Intervention Study, 11-year follow-up. Diabetologia.

[3] Muggeo M, Zoppini G, Bonora E (2000). Fasting plasma glucose variability predicts 10-year survival of type 2 diabetic patients: the Verona Diabetes Study. Diabetes Care.

[4] Jones SC, Saunders HJ, Qi W, Pollock CA (1999). Intermittent high glucose enhances cell growth and collagen synthesis in cultured human tubulointerstitial cells. Diabetologia.

[5] Schmitz O, Juhl CB, Lund S (2000). HbA1c does not reflect prandial plasma glucose excursions in type 2 diabetes. Diabetes Care.

[6] Temelkova-Kurktschiev TS, Koehler C, Henkel E, Leonhardt W, Fuecker K, Hanefeld M (2000). Postchallenge plasma glucose and glycemic spikes are more strongly associated with atherosclerosis than fasting glucose or HbA1c level. Diabetes Care.

[7] Buse JB, Freeman JL, Edelman SV, Jovanovic L, McGill JB (2003). Serum 1,5-anhydroglucitol (GlycoMark ): a short-term glycemic marker. Diabetes Technol Ther.

[8] amanouchi T, Tachibana Y, Akanuma H (1992). Origin and disposal of 1,5-anhydroglucitol, a major polyol in the human body. Am J Physiol.

[9] Akanuma Y, Morita M, Fukuzawa N, Yamanouchi T, Akanuma H (1988). Urinary excretion of 1,5-anhydro-D-glucitol accompanying glucose excretion in diabetic patients. Diabetologia.

[10] Stenninger E, Lindqvist A, Aman J, Ostlund I, Schvarcz E (2008). Continuous subcutaneous glucose monitoring system in diabetic mothers during labour and postnatal glucose adaptation of their infants. Diabet Med.

[11] Rodbard D (2009). Interpretation of continuous glucose monitoring data: glycemic variability and quality of glycemic control. Diabetes Technol Ther.

[12] McDonnell CM, Donath SM, Vidmar SI, Werther GA, Cameron FJ (2005). A novel approach to continuous glucose analysis utilizing glycemic variation. Diabetes Technol Ther.

[13] Molnar GD, Taylor WF, Ho MM (1972). Day-to-day variation of continuously monitored glycaemia: a further measure of diabetic instability. Diabetologia.

[14] Kovatchev BP, Cox DJ, Gonder-Frederick LA, Young-Hyman D, Schlundt D, Clarke W (1998). Assessment of risk for severe hypoglycemia among adults with IDDM: validation of the low blood glucose index. Diabetes Care.

[15] Yamanouchi T, Akanuma H, Asano T, Konishi C, Akaoka I, Akanuma Y (1987). Reduction and recovery of plasma 1,5-anhydro-D-glucitol level in diabetes mellitus. Diabetes.

[16] Nowatzke W, Sarno MJ, Birch NC, Stickle DF, Eden T, Cole TG (2004). Evaluation of an assay for serum 1,5-anhydroglucitol (GlycoMark) and determination of reference intervals on the Hitachi 917 analyzer. Clin Chim Acta.

[17] Shi H, Fang J, Yang X, Shen Z, Zhu X (1999). Serum 1,5-anhydro-D-glucitol as a new clinical marker for glucose metabolism in type 2 diabetics. Chin Med J (Engl).

[18] Kovatchev BP, Cox DJ, Kumar A, Gonder-Frederick L, Clarke WL (2003). Algorithmic evaluation of metabolic control and risk of severe hypoglycemia in type 1 and type 2 diabetes using self-monitoring blood glucose data. Diabetes Technol Ther.

[19] Dworacka M, Winiarska H (2005). The application of plasma 1,5-anhydro-D-glucitol for monitoring type 2 diabetic patients. Dis Markers.

[20] Dungan KM, Buse JB, Largay J (2006). 1,5-anhydroglucitol and postprandial hyperglycemia as measured by continuous glucose monitoring system in moderately controlled patients with diabetes. Diabetes Care.

[21] Nguyen TM, Rodriguez LM, Mason KJ, Heptulla RA (2007). Serum 1,5-anhydroglucitol (Glycomark) levels in children with and without type 1 diabetes mellitus. Pediatr Diabetes.

[22] Kishimoto M, Yamasaki Y, Kubota M (1995). 1,5-Anhydro-D-glucitol evaluates daily glycemic excursions in well-controlled NIDDM. Diabetes Care.

[23] Robertson DA, Alberti KGMM, Dowseb GK, Zirnmetb P, Tuomilehto J, Gareebood H (1993). Is serum anhydroglucitol an alternative to the oral glucose tolerance test for diabetes screening?. Diabet Med.

